# Optical coherence tomography and molecular analysis of sudden acquired retinal degeneration syndrome (SARDS) eyes suggests the immune‐mediated nature of retinal damage

**DOI:** 10.1111/vop.12597

**Published:** 2018-08-15

**Authors:** Sinisa D. Grozdanic, Tatjana Lazic, Helga Kecova, Kabhilan Mohan, Markus H. Kuehn

**Affiliations:** ^1^ Department of Veterinary Clinical Sciences College of Veterinary Medicine Iowa State University Ames Iowa; ^2^ Animal Eye Consultants of Iowa Hiawatha Iowa; ^3^ TL VetPath International Consultants Hiawatha Iowa; ^4^ Department of Ophthalmology and Visual Sciences Roy J. and Lucille A. Carver College of Medicine University of Iowa Iowa City Iowa

**Keywords:** canine, detachment, immune, microarray, optical coherence tomography, retina, sudden acquired retinal degeneration syndrome

## Abstract

**Objective:**

To perform detailed analysis of retinal changes in dogs with SARDS using optical coherence tomography (OCT), funduscopy, and molecular analysis.

**Animals:**

Subjects were 29 dogs from 12 US states and Canada diagnosed with SARDS by 8 ophthalmologists. An additional 7 eyes from 5 deceased SARDS dogs were used for molecular and histological analysis.

**Procedures:**

Dogs were evaluated using chromatic pupil light reflex testing (cPLR), and electroretinography (ERG); subjects underwent complete ophthalmic examination, including funduscopy, retinal photography, and OCT, in addition to complete laboratory analysis, blood pressure evaluation, abdominal and thoracic radiographs, and computerized tomography (CT) imaging to assess possible systemic abnormalities. Histology and immunohistochemistry analysis was performed in 2 SARDS eyes. Microarray analysis was performed in 5 SARDS retinas.

**Results:**

Thirty‐eight percent of patients had <1‐mm wide retinal detachments (RD) on OCT analysis, which could not be detected by funduscopy or retinal photographs. Systemic hypertension did not seem to be a contributing factor (RD 22.2%; ND 20%, Odds ratio = 1.1). No dogs showed neoplastic changes by thoracic or abdominal radiography, or CT imaging. There was no statistically significant difference in age (RD 7.9 ± 1.9 years (mean ± SD); ND 7.6 ± 1.7 years, *p* = 0.69) or duration of blindness prior to presentation (RD 18 ± 7 days (mean±SD); ND 21 ± 12 days, *p* = 0.28). Microarray and histology analysis of SARDS eyes revealed molecular changes suggestive of immune‐mediated damage.

**Conclusions:**

Observed histological, molecular, and OCT changes are highly suggestive of immune‐mediated damage in SARDS eyes.

## 1 INTRODUCTION

Sudden Acquired Retinal Degeneration Syndrome (SARDS) is recognized as one of the most frequent irreversible causes of blindness in the canine population.^1^, ^2^, ^3^ SARDS is characterized by sudden‐onset blindness, completely extinguished retinal electrical responses, and abnormal chromatic pupil light reflex (cPLR) properties (no red PLR–good blue PLR).^3^, ^4^, ^5^ It has been theorized that SARDs is an autoimmune disease similar to non‐paraneoplastic autoimmune retinopathies in humans (npAIR), with a strong autoantibody component. This hypothesis is based on limited molecular and histology data,^5^ and findings of retinal autoantibodies in serum samples of SARDs patients;^6^, ^7^ however, the lack of intraocular inflammatory changes has long been used as an argument for postulating a non‐inflammatory character for this syndrome.^1^, ^8^

Spectral domain optical coherence tomography (SD‐OCT) is an imaging method that allows high‐speed, high‐resolution cross‐sectional imaging of the retina, and optic nerve (“in vivo histology”). In recent years, it has been adopted for characterization of morphology in healthy and diseased canine retinas.^9^, ^10^, ^11^, ^12^, ^13^, ^14^, ^15^, ^16^, ^17^, ^18^, ^19^ High‐quality images can be obtained by collecting SD‐OCT of retinal cross‐sections using the infrared beam produced by a superluminescent diode. This imaging technique allows for detailed morphological mapping and structural evaluation of different retina layers and optic nerve structures in dogs.^9^

Optical coherence tomography (OCT) data in SARDS patients have thus far been reported in only few studies, which revealed significant retinal and retinal nerve fiber layer thinning (RNFL) or photoreceptor thickness in patients.^5^, ^20^ The principal purpose of this study was to provide a detailed analysis of the retina in SARDS dogs using more sophisticated technology (SD‐OCT) than the standard OCT technique that was previously available.^5^ Furthermore, we wanted to evaluate whether indirect ophthalmoscopy and fundus imaging have equal sensitivity in terms of detecting retinal changes in SARDS patients. We also wished to assess whether the presence of systemic organ abnormalities and duration of blindness affect retinal structural parameters in SARDS dogs. Finally, we wanted to investigate histological and molecular features of SARDS retina, with a particular focus on the cellular and genetic components associated with immune‐mediated tissue events, by evaluating gene expression profiles in SARDS retinas using microarray analysis, and histological and immunohistochemical (IHC) techniques with the specific goal of identifying different immune cell populations.

## 2 MATERIALS AND METHODS

All studies were conducted in accordance with the ARVO Statement for Use of Animals in Ophthalmic and Vision Research. Procedures were approved by the Iowa State University Committee on Animal Care (2‐07‐6307‐K, February 20, 2007; 5‐07‐6362‐K, June 13, 2007). A total of 29 canine clinical patients presented to Iowa State University–Veterinary Teaching Hospital were evaluated for SARDS in the period between January 2008 and December 2010. Patients were from 12 US states and Canada, and were diagnosed with SARDS by 8 different ophthalmologists. Controls were 21 healthy female beagles (6 years of age) for SD‐OCT data comparison. Eyes were collected for histology analysis from two SARDS patients euthanized within 4 weeks of SARDS diagnosis outside of Iowa State University; patients were diagnosed with SARDS based on the clinical presentation of sudden‐onset blindness, near normal retinal appearance (vascular attenuation only) and completely extinguished ERG responses. Both patients were euthanized due kidney failure (7‐year‐old castrated male [CM] poodle and 6‐year‐old CM Maltese). Five eyes from 3 SARDs patients were used for microarray analysis (all patients were diagnosed with SARDS based on a history of sudden‐onset vision loss, completely extinguished ERG responses, and absent chromatic pupil light reflex response to red and positive response to blue light): (1) Maltese–8‐year‐old CM, died due to kidney and cardiac failure 19 months after SARDS diagnosis; (2) Pug–9‐year‐old CM, died 30 months post SARDS diagnosis due to the severe intestinal disease, pancreatitis, and liver failure; (3) Pug–8‐year‐old CM, died 8 months post SARDS diagnosis due to neurological problems (neuronal necrosis and diffuse chronic myositis diagnosed on histopathology with minimal inflammatory changes). Patients 1 and 2 were not on immunosuppressive medications at the time of euthanasia, while patient 3 was receiving systemic cyclosporine at the time of euthanasia. One eye from patient 3 was used for the microarray analysis, while the other eye was used for the IHC and histology analysis. An additional 5 control eyes from 5 healthy control dogs (Beagle, SF, 6 years old) without evidence of ocular abnormalities were used for microarray analysis. Eyes for microarray analysis were collected immediately upon euthanasia, and retinal tissue was collected and stored in RNAlater (Ambion Inc., Foster City, CA). Retinal samples stored in the RNAlater solution were frozen at −80°C until RNA isolation for microarray experiments.

### 2.1 Diagnosis of SARDS

Diagnosis of spontaneously occurring SARDS in dogs was confirmed at Iowa State University based on previously established criteria^4^: history of sudden onset of blindness, normal or almost completely normal fundus appearance, normal intraocular pressure, absent PLRs with red‐light stimuli (630 nm, 5.3 log units intensity), normal PLRs with blue‐light stimuli (480 nm, 5.3 log units intensity), and completely extinguished ERG responses. All SARDS patients and healthy control dogs received a complete ophthalmic examination: slit lamp biomicroscopy, indirect ophthalmoscopy, tear production, and intraocular pressure evaluation, as well as a basic neuro‐ophthalmology evaluation (palpebral and corneal reflex, ocular motility evaluation). Menace, dazzle, PLRs, and visual maze testing was performed to evaluate status of the visual system. Diagnosis of SARDS in two euthanized dogs was performed based on completely extinguished ERG responses, near normal retinal appearance and historical evidence of sudden‐onset blindness prior to euthanasia. Chromatic pupil light reflex data were not available for euthanized patients. Full necropsy was not performed on euthanized patients (only eyes were collected).

### 2.2 Systemic SARDS evaluation

All patients also underwent complete cell blood count and serum chemistry, urine analysis (22 of 29, all samples were collected via cystocentesis), systolic blood pressure (SBP) evaluation (19 of 29, SBP was evaluated with an ultrasonic Doppler flow detector, Model 811‐L, Parks Medical Electronics Inc., Las Vegas, NV, USA), and thoracic and abdominal radiographs (29 of 29). In 15 of 29 patients, brain computerized tomography (CT) imaging was performed. Brain CT imaging was conducted prior to the referral to Iowa State University, or during the visit. Abdominal and thoracic radiographs, and brain CT images were reviewed by board certified radiologists and residents at respective institutions where imaging was performed. Only head CT imaging was performed (no thorax or abdomen CT imaging was pursued).

### 2.3 Functional retinal evaluation in vivo

Pupil light reflex, fundus photography, and electroretinography was performed in clinical and experimental dogs, as described previously.^4^, ^5^, ^21^ OCT analysis in canine patients was also performed as previously described.^9^

### 2.4 The pupil light‐reflex analysis

Analysis of the cPLR response in clinical and healthy experimental dogs was performed using a Melan‐100 unit (BioMed Vision Technologies, Ames, IA) with the goal of differentiating rod‐cone vs melanopsin (intrinsically photosensitive retinal ganglion cells–ipRGCs), as previously reported.^4^, ^22^ The Melan‐100 unit has a diode‐based light source with a narrow wavelength: blue light (480 nm, 5.3 log units intensity) and red light (630 nm, 5.3 log units intensity). Pupil diameter values were measured as reported previously.^22^

### 2.5 Electroretinography

Electroretinography was used to evaluate retinal function in SARDS dogs (n = 29). A Roland Consult ERG system (Roland Consult, Brandenburg, Germany) and Retinographics ERG system (Retinographics, Norwalk, CT) were used to deliver light stimuli and collect signals from the lens electrode for full‐field ERG recording routines, as previously reported.^5^, ^21^

### 2.6 Fundus photography

Fundus photography was performed using RetCam Fundus Camera system (Massie Research Laboratories, Pleasanton, CA) as previously reported.^5^

### 2.7 Optical coherence tomography

Optical coherence tomography analysis of different retinal layer thicknesses was performed as previously reported using a Heidelberg Engineering Spectralis OCT unit (Heidelberg Engineering, CA, USA).^9^ The following scans were obtained and analyzed: peripapillary circle scan; horizontal volume scan through *area centralis* (within the visual streak; located dorso‐temporally from the optic nerve head) in the superior‐temporal (tapetal) retina; and a corresponding volume scan in the ventrotemporal (non‐tapetal) retina. Additional horizontal volume scans were performed based on the funduscopic evidence of possible retinal lesions with a focus on hyperpigmented, hyper‐reflective, hypopigmented, and potentially exudative lesions.

### 2.8 Histology analysis

Three eyes from SARDS patients and 5 eyes from healthy control dogs were fixed in 10% formalin. Eyes were embedded in paraffin and 7‐μm tissue sections were prepared. A total of 10 retinal sections of central (temporal and nasal) and peripheral (temporal and nasal) retina were evaluated for each eye. Standard hematoxylin and eosin stain was performed and slides were coverslipped. Tissue sections were examined under a photomicroscope (Microphot FXA; Nikon, New York, NY). Images were captured using a camera (Megaplus, model 1.4; Eastman Kodak, Rochester, NY) connected to a frame grabber (MegaGrabber; Perceptics, Knoxville, TN) in a computer (Macintosh 8100/80 AV; Apple Computer, Cupertino, CA) using image acquisition and analysis software (Metamorph; Molecular Devices, Sunnyvale, CA).

### 2.9 Immunohistochemistry analysis

Immunohistochemistry IHC analysis on canine retinal tissue was performed as previously reported.^23^ Briefly, tissue samples for IHC were fixed in 4% paraformaldehyde, embedded in paraffin and sectioned into 5‐7 μm thick sections. Sections were deparaffinized with heat and xylene, and rehydrated by serial rinses in decreasing concentrations of ethanol. Endogenous peroxidase activity was quenched by incubation with 3% H_2_O_2_ for 10 minutes. Following rinses in potassium phosphate‐buffered saline (KPBS), cells were incubated in blocking solution containing 5% normal donkey serum (NDS, 017–000–121; Jackson ImmunoResearch, West Grove, PA), 0.1% BSA (BSA, A9647; Sigma, St. Louis, MO), and 0.04% Triton X‐100 for 2 hours to eliminate non‐specific antibody labeling. Tissue was then incubated in primary polyclonal antibodies overnight at room temperature including: anti‐CD3 (T‐lymphocyte marker; Dako, Carpinteria, CA); anti‐CD79 (B‐lymphocyte marker; Dako); anti‐CD18 (macrophage marker; Dako), and a cocktail of IgG, IgM and IgA for detection of immunoglobulin producing plasma cells (Dako). Sections were then incubated with a biotinylated secondary antibody (10 minutes), and this complex was labeled with streptavidin–horseradish peroxidase conjugate and identified with diaminobenzidine, followed by Mayer's hematoxylin counterstain. Stained tissue sections were scanned using a histopathology microscope scanner (Panoramic Desk, 3DHisttech, Budapest, Hungary). Grading of IHC slides was not pursued due to the focal nature of IHC positive cells.

### 2.10 Microarray analysis

Microarray analysis on canine retinal tissue was performed as previously reported.^23^ Briefly, 5 eyes from 3 SARDs patients and 5 control eyes from healthy controls without evidence of ocular abnormalities were used for microarray analysis as follows. Eyes were dissected and preserved in RNAlater (Ambion, Austin, TX) immediately after enucleation. Samples were then stored at −80°C until RNA extraction. The neural retina was isolated, and total RNA was extracted from the tissue using Qiagen RNeasy minipreps. Samples were treated with RNase free DNase, and the integrity of the RNA was evaluated through analysis with a Bioanalyzer (Agilent Technologies, Foster City, CA). The range of and a mean value for RNA quality (RIN) for patient and control samples was as following: mean RIN = 8.75, Range: 8.6‐9.0. RNA was amplified using a T7 RNA polymerase‐based approach, and hybridized to Affymetrix Canine genome 2.0 gene chips following standard protocols.^23^

Obtained raw data were normalized using the RMA algorithm. Normalized data were log2‐transformed and filtered to remove non‐expressed genes from the dataset. For the purpose of this study, expressed genes were defined as those with corresponding probe sets displaying log‐expression values above 7.0 in at least 2 samples (either controls or affected). The remaining probe sets were analyzed to identify significant expression changes using the Wilcoxon unpaired rank sum test and the significance analysis for microarray (SAM; Version 3.0; Microsoft Excel Add‐In, Stanford University, Stanford, CA). Data were analyzed 4 times using 200 permutations and different seeds values for the random number generator. The delta value was set at 0.53, and a minimum two‐fold expression change was required. Only genes identified as differentially expressed in all 4 analyses are presented in this manuscript.

### 2.11 Statistical analysis

Statistical analysis between groups was performed using unpaired Student's *t* test. Fischer's exact test and strength of association between tested parameters (odds ratio [OR]) calculations were performed between different observed parameters as indicated with commercial software (Prism, version 5.0; GraphPad, San Diego, CA). Statistical significance was set at *P* < 0.05. Prior to statistical analysis for Student's *t* test, data were converted using the log function to provide normalization of data distribution. The odds ratio is the ratio with and without an event in each group; it indicates the probability that an event will occur, divided by the probability that an event will not occur in association with the specific parameter.

## 3 RESULTS

### 3.1 Patient population

Females were more prevalent (20 of 29 patients; 69%; Table 1) in the SARDS population, compared to males (9 of 29 patients; 31%). The mean age for the population was 7.7 ± 1.8 years (mean ± SD, median value = 7 years; range 5–11 years). The Dachshund breed was most prevalent (6/29; 20.7%), followed by the Miniature Schnauzer (5/29;17.2%), Maltese (3/29; 10.3%) and mixed‐breed dogs (3/29; 10.3%).

**Table 1:** Breed, age, and sex distribution of SARDS patients with geographic location and clinical findings on indirect ophthalmoscopy and optical coherence tomography

	Breed	State	Sex	Age (y)	Duration of vision loss (d)	ERG	cPLR	Fundus changes (indirect ophthalmoscopy)	OCT lesions
1	Mix	GA	SF	10	60	Flat	NR‐GB	pONH, VA, HPS	D, OSD
2	Dachshund	ND	SF	7	21	Flat	NR‐GB	pONH, VA, HPS	D, T, CHRS, OSD
3	Pug	TX	F	5	10	Flat	NR‐GB	pONH, VA, ATR	D, T, CHRS, OSD
4	Dachshund	FL	SF	6	7	Flat	NR‐GB	PEL	D, OSD
5	West High. Ter.	MN	SF	10	7	Flat	NR‐GB	ATR	D, OSD
6	Springer Spaniel	NC	SF	9	10	Flat	NR‐GB	ATR	D, T, OSD
7	Maltese	MN	SF	7	3	Flat	NR‐GB	WNL	D, OSD
8	Dachshund	MN	SF	7	28	Flat	NR‐GB	pONH, VA, ATR	D, OSD
9	Dachshund	WA	SF	11	21	Flat	NR‐GB	pONH, VA, ATR	D, OSD
10	Beagle	NC	M	6	2	Flat	NR‐GB	VA, PEL,	D, CHRS, OSD
11	Mixed	MN	CM	9	28	Flat	NR‐GB	HR‐PV,	D, T, OSD
12	Maltese	CA	CM	9	60	Flat	NR‐GB	pONH, VA, ATR	OSD
13	Mixed	MN	SF	9	30	Flat	NR‐GB	pONH, VA, ATR	OSD
14	Beagle	FL	CM	6	30	Flat	NR‐GB	pONH, VA, HR‐PV, HPS	OSD
15	Chihuahua	TX	SF	10	28	Flat	NR‐GB	pONH, VA, FH	T, OSD
16	Min Schnauzer	NV	SF	10	30	Flat	NR‐GB	pONH, VA	OSD
17	Lab. Retriever	MN	F	10	14	Flat	NR‐GB	PEL, PRE	OSD
18	Min Schnauzer	IN	CM	7	14	Flat	NR‐GB	pONH, PRE, VA, ATR	OSD
19	Min Schnauzer	IL	CM	5	14	Flat	NR‐GB	HR‐F, PEL	CHRS, OSD
20	Min Schnauzer	MN	CM	11	28	Flat	NR‐GB	pONH, VA, ATR	OSD
21	Jack Russel Ter.	CAN	SF	6	21	Flat	NR‐GB	pONH, VA, HR‐F, ATR	T, OSD
22	Dandie Diam. Ter.	OR	F	7	21	Flat	NR‐GB	pONH, VA	T, OSD
23	Lab. Retriever	IA	SF	9	21	Flat	NR‐GB	ATR	OSD
24	Britany Spaniel	MN	SF	7	21	Flat	NR‐GB	ATR	OSD
25	Min Schnauzer	CAN	SF	6	14	Flat	NR‐GB	VA (mild), ATR	T, OSD
26	Dachshund	IL	SF	6	14	Flat	NR‐GB	ATR, HR‐D	OSD
27	Maltese	CAN	SF	8	14	Flat	NR‐GB	VA	OSD
28	Pomeranian	IA	CM	7	14	Flat	NR‐GB	VA	OSD
29	Dachshund	CA	CM	6	21	Flat	NR‐GB	pONH, VA	OSD

NR‐GB (no red, good blue).

ATR, altered tapetal reflectivity (“tapetal ridging”); CHRS, chorioretinal scar; D, retinal detachment; FH, focal hemorrhage; HPS, hyper‐pigmented spot; HR, hyper‐reflectivity (HR‐F, focal; HR‐PV, perivascular; HR‐D, diffuse); OSD, outer segment disorganization; pONH, pale optic nerve head; PEL, potentially perivascular exudative lesion; PRE, perivascular retinal edema; T, severe retinal thinning; VA, vascular attenuation; WNL, within normal limits.

Legend for OCT changes:

Patients diagnosed with SARDS originated from the following states (Table 1): Minnesota (8/29; 27.5%), Canada (3/29; 10.3%), California/Texas/North Carolina/Illinois/Iowa (2/29; 7%), Florida/Nevada/Georgia/Washington/Indiana/Oregon/North Dakota (1/29; 3%). The median time between onset of vision loss and presentation to an ophthalmologist was 20.9 ± 13.8 days (Table 1).

### 3.2 Functional analysis of SARDS patients

All evaluated patients had completely extinguished retinal electrical responses (“flat ERG”), and characteristic chromatic pupil light‐reflex deficits resulting in absent constriction after red‐light illumination, together with complete pupil constriction after blue‐light illumination (Table 1).

### 3.3 Fundoscopic findings in SARDS patients

**Figure 1:**
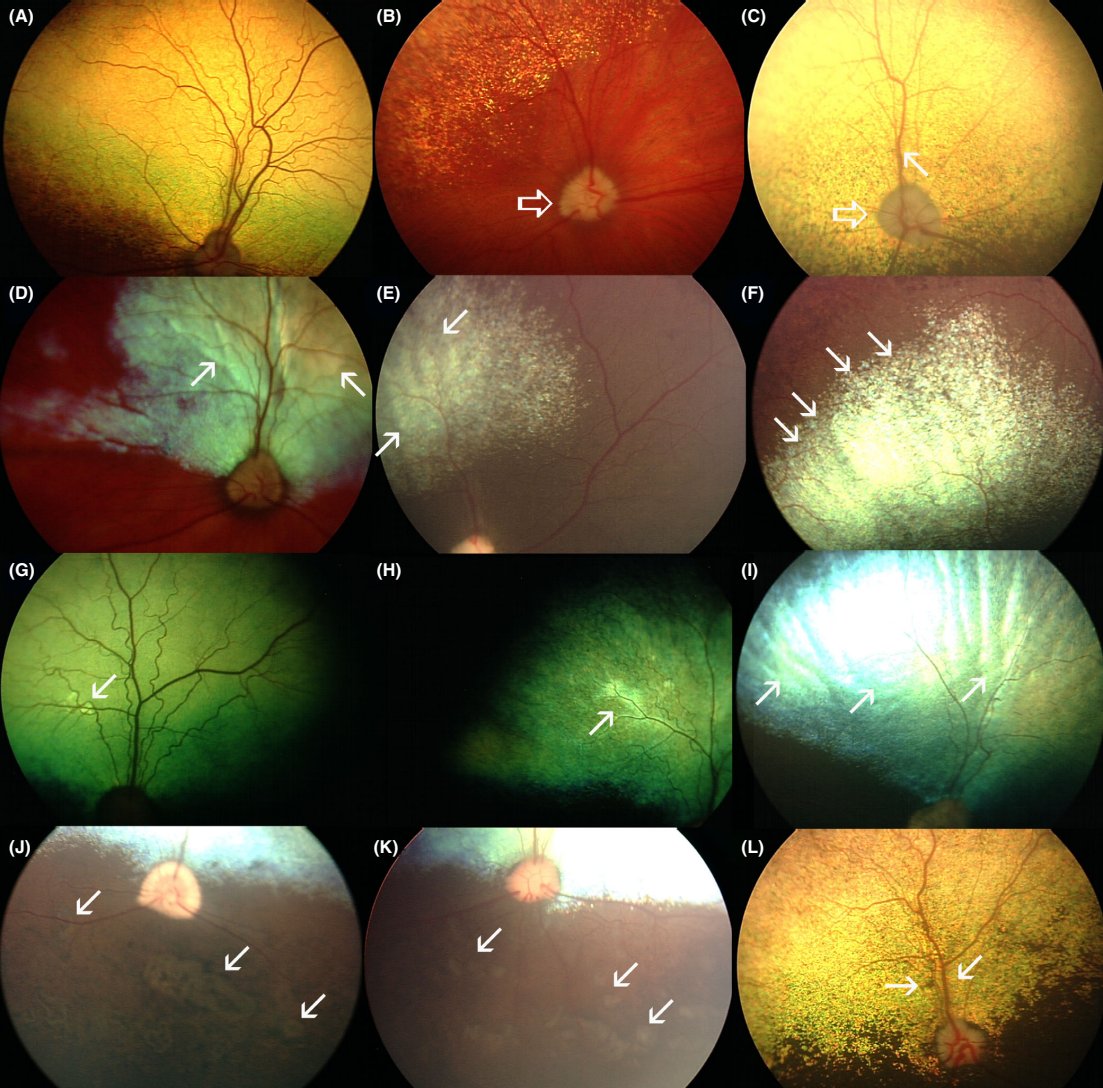
A, Normal fundus appearance in SARDS patient (3 wk after onset of blindness, Brittany Spaniel, castrated male‐CM, 6 y old). This patient was not part of this study. B, Pale optic nerve head appearance (arrow) was a frequent finding in SARDS patient population (Chihuahua, spayed female—SF, 10 y old, patient no 15). C, Pale optic nerve (open arrow) and vascular attenuation (closed arrow) were also frequent fundus finding in SARDS patients (Maltese, CM, 9 y old, patient no 12). D, Alternated tapetal reflectivity can be observed in SARDS eyes as gray field‐like zones (arrows on image D, Jack Russel Terrier, SF, 6 y old, patient no 21) or gray linear zones (arrows on image E, Dachshund, SF, 7 y old, patient no 8). F, Peripheral tapetal ridging (arrows) can be seen as a result of decreased retinal thickness. This image is overexposed to better show this type of funduscopic change (no true hyperreflectivity was observed, Dachshund, SF, 7 y old, patient no 8). G, Focal and very subtle perivascular reflectivity (arrow) can be observed in SARDS retina (Mixed, CM, 9 y Old, patient no 11). H, Linear diffuse perivascular hyperreflectivity (arrow) was observed in this patient (Dachshund, SF, 6 y Old, patient no 26‐OD). I, Diffuse hyperreflectivity (arrows) is a very rare finding in SARDS patients during the early course of disease; However, in this patient (Dachshund, SF, 6 y old, patient no 26‐OS) There was asymmetric disease appearance with the right eye being less affected (image H). (J,K), Perivascular lesions (arrows) can have potentially exudative/transudative appearance and may be associated with retinal thinning and focal retinal detachments. Image J is patient no 26 (Dachshund, SF, 6 y old), while image K is from patient no 11 (Mixed breed, CM, 9 y old). L, Perivascular retinal edema (oblique arrow) and perivascular chorioretinal scar (horizontal arrow) in SARDS patient (Mixed breed, SF, 10 y old, patient no 1)

Fundoscopic evaluation of SARDS patients revealed presence of various changes. The most frequently observed fundus change was vascular attenuation (VA) resulting in decreased thickness of blood vessels, and sporadic loss of tertiary retinal vein branches, which was observed in 65% of patients (19/29; Table 1; Figure 1B,C). In addition, pale optic nerve head appearance (pONH) was observed in 51.7% of patients (15/29; Table 1; Figure 1B,C). Altered tapetal reflectivity can be observed in SARDS eyes as gray field‐like zones (Figure 1D) or gray linear zones (Figure 1E), which change in intensity with angle of illumination, but never have true hyper‐reflective or hypo‐reflective appearance. Altered tapetal reflectivity with or without evidence of peripheral “tapetal ridging” (Figure 1F) was found in 48.2% of patients (14/29; Table 1; Figure 1D,E). Hyper‐reflective lesions were present in 17% of patients (5/29; Table 1; Figure 1G,H,I), and potentially perivascular exudative lesions (PEL) were present in 14% of patients (4/29; Table 1; Figure 1J,K). Hyperpigmented spots (HPS) were present in 10% of patients (3/29; Table 1; Figure 1L), while perivascular retinal edema (PRE) was present in 6.9% of patients (2/29; Table 1).

### 3.4 Optical coherence tomography analysis in SARDS patients

#### 3.4.1 Comparison of fundus lesions and OCT findings

**Figure 2:**
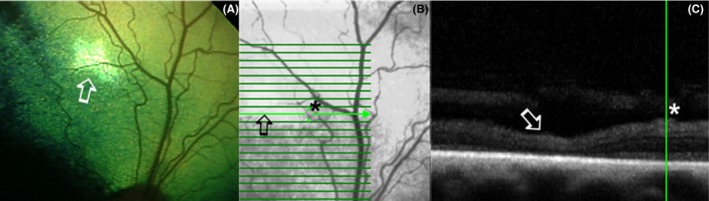
A, Perivascular retinal degenerative changes presenting as perivascular zones of hyperreflectivity (arrow) in SARDS (Mixed breed, CM, 9 y old, Table 1, patient no 11). B, Infrared fundus image of the same patient. Arrow points to the linear scan through hyperreflective zone, star marks the position of the blood vessel and vertical OCT scan marker. C, Perivascular focal retinal degenerative changes (arrow) with primary loss of photoreceptors can be observed on OCT scan in SARDS patient. Star marks the position of the blood vessel, and the green line indicates the position of the vertical OCT scan marker

**Figure 3:**
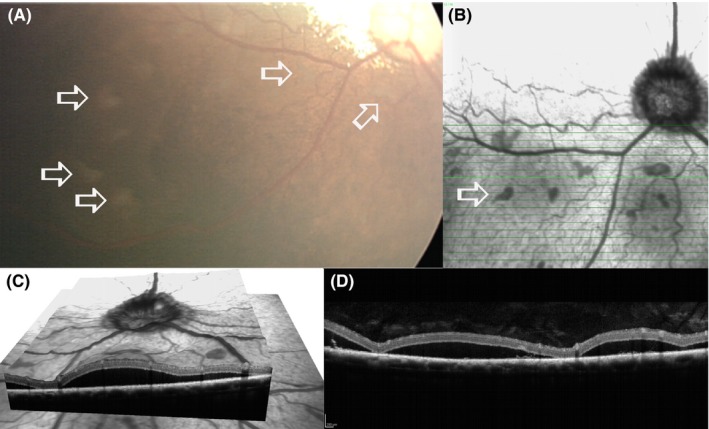
A, Potentially exudative lesions could be detected in SARDS patients (arrows) as whitish or gray focal zones. These lesions were most frequently detected in perivascular spaces. initially this lesion was described as a normal fundus variation during indirect ophthalmoscopy (Mixed breed, CM, 9 y old, Table 1, patient no 11). B, Infra‐red image fundus of the same region revealed more prominent appearance of lesions. C, 3D OCT Reconstruction of the same region showing bullous retinal detachments of approximately 200‐400 μm thickness. D, Linear OCT scan through the region of interest shows multiple bullous retinal detachments in this SARDS patient

**Figure 4:**
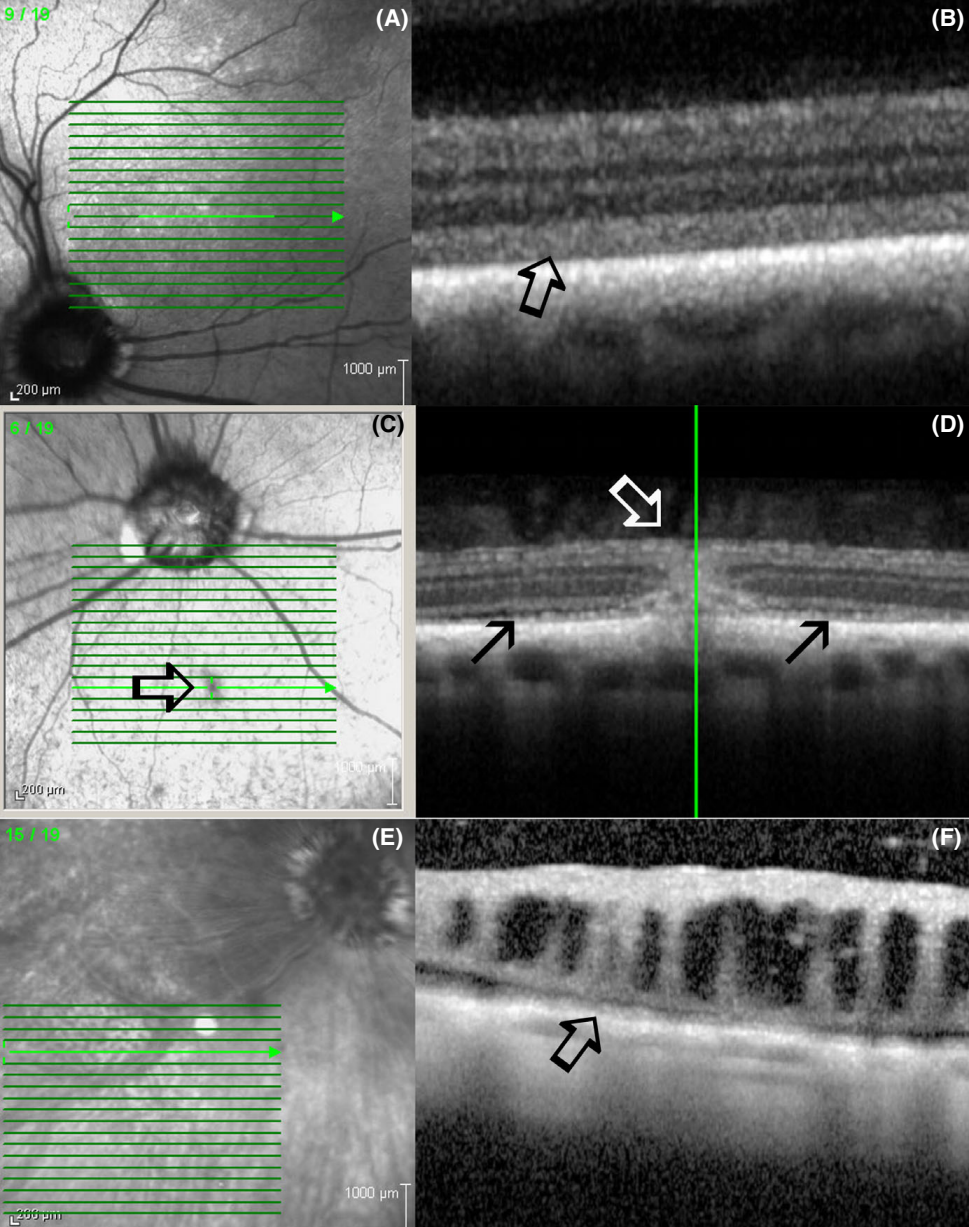
A,B, Alternated tapetal reflectivity zones are always associated with disorganization of inner and outer photoreceptor segments, resulting in the loss of stratification (arrow, Labrador Retriever, F, 10 y old, Table 1, patient no 17). (C,D), Hyperpigmented spots can be observed in SARDS eyes and can be associated with chorioretinal scars on OCT Scans (4C, Pug, F, 5 y Old, Table 1, patient no 3) or zones of retinal detachment (as shown in image 3B,C,D). (E,F), Potentially perivascular exudative lesions and perivascular retinal edema can be associated with retinal detachment as shown in image 3, or potentially exudative process (arrow) with cystic retinal degeneration as shown in this SARDS patient (Dachshund, SF, 11 old, Table 1, patient no 9)

Optical coherence tomography analysis and comparison with fundus images revealed that zones of hyper‐reflective appearance were associated with focal retinal structural photoreceptor loss (Figure 2). Zones of PEL were associated with potentially exudative retinal detachments, retinal edematous, and cystic changes or chorioretinal perivascular thinning (Figures 3 and 4E,F). Altered tapetal reflectivity was associated with disorganization of inner and outer photoreceptor segments, resulting in the loss of stratification (Figure 4A).

### 3.5 Optical coherence tomography thickness parameters in SARDS patients

Detailed analysis of different thickness parameters revealed mild but significant loss of total retinal thickness in SARDS canine patients in the superio‐temporal (*area centralis* region) retina (*P* = 0.04, Student's *t* test; Table 2). Much more prominent damage was observed in the corresponding inferior retina region (*P* = 0.0001, Student's *t* test) when subjects were compared to 6‐year‐old healthy control dogs. Evaluation of the receptor layer thickness (combined thickness of outer nuclear layer and inner and outer segment layer) revealed significant loss in SARDS dogs in the *area centralis* (*P* < 0.0001, Student's *t* test; Table 2) and corresponding inferior retina (*P* < 0.0001, Student's *t* test) compared to healthy control dogs.

**Table 2:** Optical coherence tomography analysis of SARDS dogs

		Retinal thickness	ONL+IS+OS thickness	ONL thickness	RNFL
ST	SARDS	190 ± 9^a^	66.3 ± 1.9^c^	43.2 ± 1.6^a^	28.6 ± 1
	CTRL	198.2 ± 2	95.5 ± 1.8	50.1 ± 1.8	26.2 ± 0.3
IT	SARDS	148 ± 2.9^c^	53.4 ± 1.9^c^	38.9 ± 1.1^b^	23.9 ± 0.8
	CTRL	163.9 ± 1.4	78.7 ± 2.1	44.3 ± 1.1	24.9 ± 0.4

CTRL, healthy control dogs; IS, inner segments; IT, inferior temporal region; ONL, outer nuclear layer; OS, outer segments; RNFL, retinal nerve fiber layer; ST, superior temporal retinal region (area centralis).

Optical coherence tomography revealed significant thinning of outer nuclear layer, particularly outer segments in SARDS patients compared to healthy control dogs.

All values are expressed as mean ± SEM (μm).

a *P *<* *0.05.

b *P *<* *0.001.

c *P *<* *0.0001

Analysis of the outer nuclear layer thickness revealed mild but significant structural loss in the superior retina (*P* < 0.02, Student's *t* test), and significantly more prominent inferior retina structural loss (*P* = 0.001, Student's *t* test). Analysis of the retinal nerve fiber layer (RNFL) in the *area centralis* revealed lack of structural loss in the superior‐temporal retina of SARDS dogs (*P* = 0.21, Student's *t* test). Similar findings were observed when inferior retina RNFL thickness was compared between SARDS and healthy control dogs (*P* = 0.12, Student's *t* test).

Analysis of peripapillary RNFL thickness did not reveal significant differences in analyzed quadrants when values were compared between SARDS and healthy control dogs (Table 3): RNFL temporal (*P* = 0.4, Student's *t* test); RNFL superior (*P* = 0.8, Student's *t* test); RNFL nasal (*P* = 0.8, Student's *t* test); and RNFL inferior (*P* = 0.64, Student's *t* test).

**Table 3:** Optical coherence tomography of peripapillary retinal nerve fiber layer (RNFL) regions in SARDS dogs

	RNFL Superior	RNFL Inferior	RNFL Temporal	RNFL Nasal
SARDS	92.1 ± 3.4	51.6 ± 2.3	80.9 ± 3.6	81.7 ± 5.4
CTRL	90.4 ± 1.5	55.2 ± 2.4	75.5 ± 2	77.7 ± 3.3

No significant difference was observed in RNFL thickness between SARDS and healthy control dogs in any of evaluated quadrants.

All values are expressed as mean ± SEM (μm).

### 3.6 Optical coherence tomography detection of retinal lesions in SARDS patients

**Figure 5:**
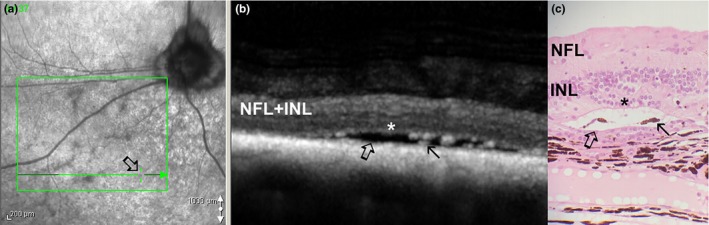
Focal micro‐retinal detachments (RD) were not detectable during fundus evaluation and in fundus photography; however, these RDs were frequently detected with the use of OCT imaging in SARDS eyes. A, Infrared fundus image of the retinal region evaluated by OCT revealed some vascular attenuation but no RD (Mix breed, SF, 10 y, Table 1, patient no 1). Detailed image analysis did not reveal possible presence of RD (arrow points to the region where focal RD was detected using OCT). B, OCT of the SARDS retina shows micro‐RD (open arrow) with photoreceptor loss (star), and most likely cellular infiltration of inflammatory cells in the RD space (closed arrow). C, Retinal histology micrograph from a SARDS retina (not the same patient as in A and B) shows micro‐RD (open arrow), photoreceptor loss (star), and pigmented macrophage infiltration in the retinal detachment space (closed arrow), confirming potentially exudative nature of retinal detachment. INL, inner nuclear layer; NFL, nerve fiber layer

**Figure 6:**
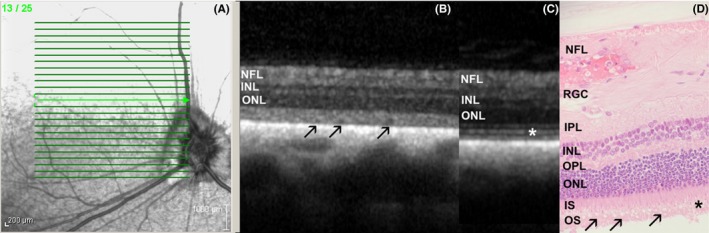
Photoreceptor outer segment disorganization was the most frequently observed optical coherence abnormality detected in SARDS patients. A, Infrared fundus image of the area centralis region evaluated by optical coherence tomography shows normal retinal appearance (Beagle, CM, 6 y old, Table 1, patient no 14). B, OCT of the SARDS retina shows loss of IS‐OS stratification and inner‐outer segment disorganization with formation of vacuolar changes (arrows). C, OCT scan of healthy canine retina‐stratification and delineation between outer and inner segments of photoreceptors is visible (star). D, Retinal histology micrograph from a SARDS retina (not from the patient in images A and B) shows outer segment disorganization (star) and severe vacuolization (arrows). INL, inner nuclear layer; IPL, inner plexiform layer; IS, inner segments; ONL, outer nuclear layer; OPL, outer plexiform layer; OS, outer segments; NFL, nerve fiber layer; RGC, retinal ganglion cells

**Figure 7:**
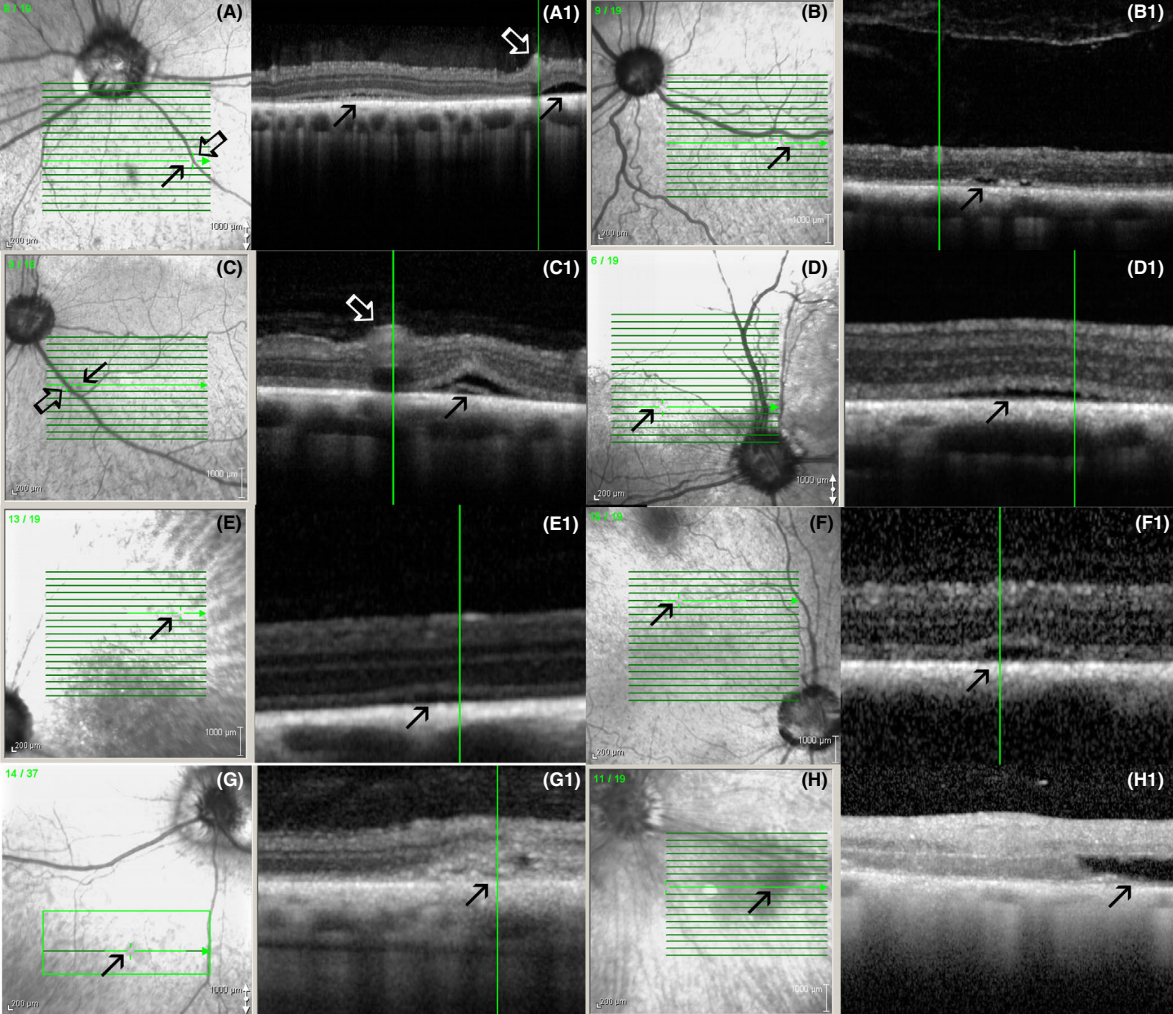
Focal micro‐retinal detachments (closed arrows) were frequently present in SARDS patients. These micro‐retinal detachments were most frequently present in perivascular spaces (open arrows point to blood vessels). Green vertical lines correspond to the retinal region shown on a linear scan. A, Patient no 3 (Table 1, Pug, F, 5 y old); B, patient no 2 (Dachshund, SF, 7 y old); C, patient no 4 (Dachshund, SF, 6 y old); D, patient no 5 (West Highland Terrier, SF, 10 y old); E, patient no 7 (Maltese, SF, 7 y old); F, patient no 8 (Dachshund, SF, 7 y old); G, patient no 10 (Beagle, M, 6 y old); H, patient no 9 (Dachshund, SF, 11 y old)

**Figure 8:**
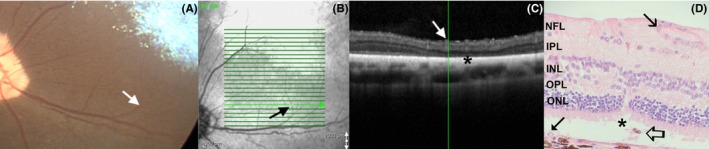
Focal outer nuclear layer loss is frequently observed by OCT in SARDS patients. A, Color fundus camera image of the evaluated SARDS patient (Pug, Female, 5 y old, Table 1, patient no 3) did not reveal any abnormalities in area of interest (arrow). B, Infrared fundus image of the retinal region evaluated by OCT (arrow points to the perivascular region of interest with focal retinal structural loss. C, OCT of the SARDS retina shows focal photoreceptor loss (star). D, Retinal histology micrograph from a SARDS retina (not the patient in images A, B and C) shows focal photoreceptor loss (star) and pigmented macrophage or activated RPE cell infiltration (open arrow). Closed tip arrow points to the non‐pigmented RPE cell in the tapetal retina. These changes were most frequently observed in perivascular spaces (closed arrow points to the blood vessel). INL, inner nuclear layer; IPL, inner plexiform layer; NFL, nerve fiber layer; ONL, outer nuclear layer; OPL, outer plexiform layer

**Figure 9:**
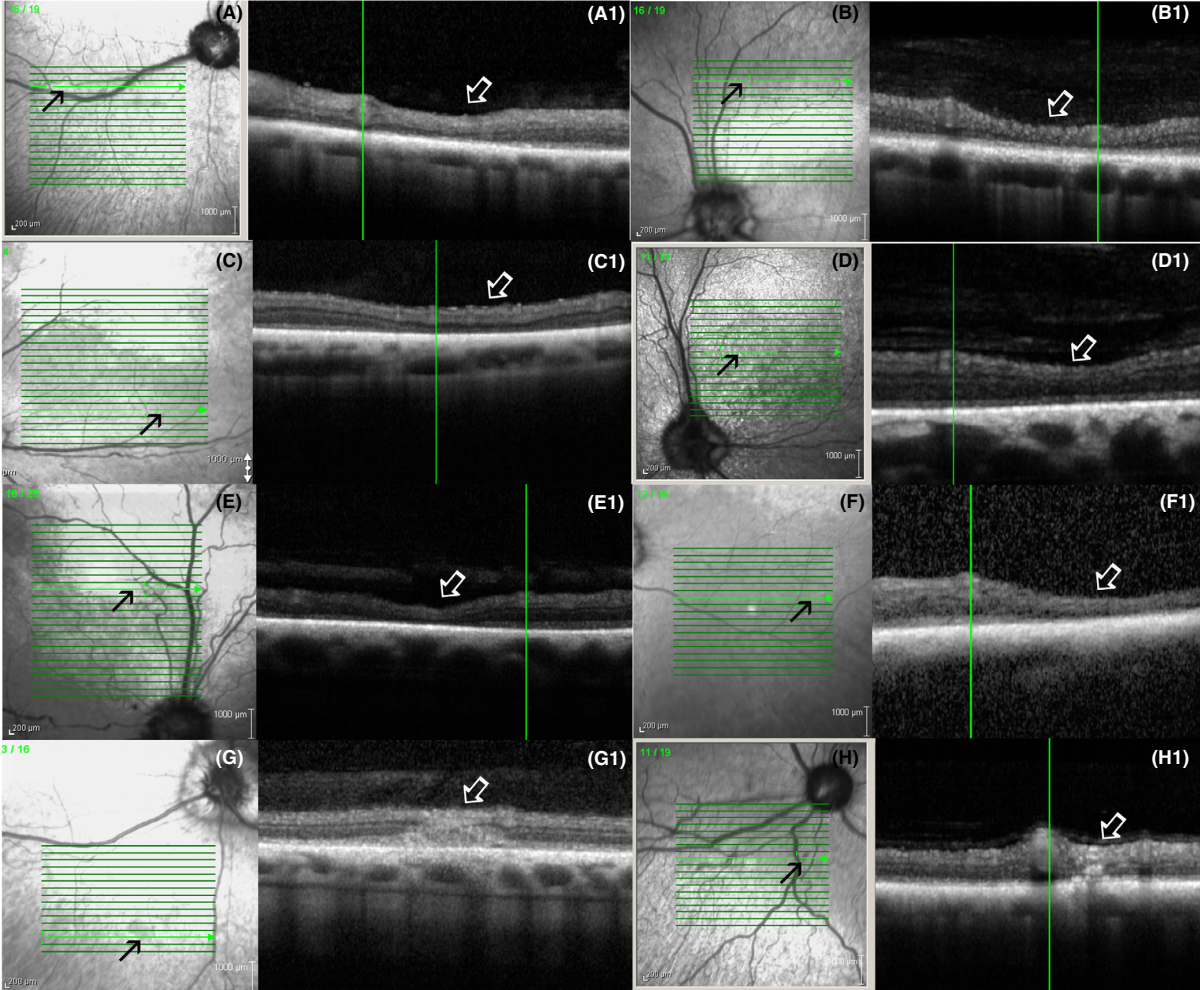
Focal perivascular zones of photoreceptor loss (A‐F) and chorioretinal scars (G‐H) were frequently observed in SARDS patients (arrows). Green vertical lines correspond to the retinal region shown on a linear scan. A, Patient no 2 (Table 1, Dachshund, SF, 7 y old); B, patient no 3 (Pug, F, 5 y old); C, patient no 21 (Jack Russel Terrier, SF, 6 y old); D, patient no 6 (Springer Spaniel, SF, 9 y old); E, patient no 11 (Mixed breed, CM, 9 y old); F, patient no 22 (Dandie Diamond Terrier, F, 7 y old); G, patient no 3 (Pug, F, 5 y old); H, patient no 2 (Dachshund, SF, 7 y old)

The most frequently observed OCT change in SARDS patients was disorganization of the outer segments of photoreceptors with a loss of the inner‐outer segment (IS‐OS) junction, which was observed in all (100%; 29/29) evaluated patients (Figures 4A and 5; Table 1). Retinal detachment (RD) was present in 38% of patients (11/29; Figures 3, 6 and 7; Table 1), severe focal retinal thinning (T) in 28% (8/29; Figures 8 and 9; Table 1), and focal chorioretinal scars (CHRS) in 14% (4/29; Figures 4C,D and 9G,H). A total of 55% of patients (16/29) had evidence of one or more OCT changes (RD, T, CHRS, Table 1).

### 3.7 Optical coherence tomography detection of retinal detachment in SARDS patients

As mentioned above, 38% (11/29) of SARDS patients (Table 1) had evidence of <1‐mm wide RD on OCT analysis, which could not be detected during funduscopic evaluation or analysis of retinal photographs (Figures 6 and 7). The Dachshund breed was over‐represented in the RD group (36%, 4/11), compared to the non‐detachment (ND) group (11%, 2/18), though this did not reach statistical significance, likely due to the very small sample size (*P* = 0.16; OR = 4.6). Females were more frequently affected with RD (9/11, 82%) compared to males (2/11, 18.2%); however, this did not reach statistical significance in Fischer's exact test (*P* = 0.4; OR = 2.8).

### 3.8 Systemic changes in SARDS patients

Analysis of metabolic changes (increased appetite/polyphagia, weight gain, polydipsia/polyuria) was performed based on historical information obtained from owners, referring veterinarians, and a review of patient medical records. Clinical signs of polydipsia/polyuria (PU/PD), polyphagia (PP) and weight gain (WG) were observed in 45% of patients (13/29; Table 4). Evaluation of serum chemistry revealed elevation of serum alkaline phosphatase (SAP) and/or alanine aminotransferase (ALT) in 52% of patients (15/29). Clinical signs of PU/PD/PP/WG and concurrent elevation of SAP/ALT were identified in 21% of patients (6/29), while 31% (9/29) had elevation of SAP/ALT but no historical or clinical signs of PU/PD/PP/WG.

**Table 4:** Laboratory and systemic abnormalities in SARDS patients

	PU/PD/PP/WG	Serum	Urine	CBC	BP	Allergic/Autoimmune disease	Other systemic changes	Systemic medications at the time of laboratory analysis
1	None	↑SAP, ↑ALT a	Proteinuria	Lymphopenia	115	Food	None	Prednisone
2	None	↑SAP, ↑ALT	N/A	None	145	None	Hepatomegaly	None
3	None	↑SAP, ↑ALT	N/A	Anemia	150	None	Cardiomegaly	None
4	None	WNL	None	None	200	Atopy	Hepatomegaly left atrial enlargement	None
5	None	↑SAP, ↑ALT^a^	Microalbuminuria	None	WNL	Atopy	None	Prednisone
6	Polyphagia, weight gain 6 mo	↑ALT	None	Leukopenia	170	None	None	Glucosamine, carprofen as needed
7	None	↑SAP	Proteinuria	None	110	None	None	None
8	Weight gain 3 mo	None	Proteinuria	None	155	N/A	None	Doxycycline, Prednisone (as needed) ‐ given 2 wk prior
9	None	None	N/A	None	N/A	N/A	Hepatomegaly	Carprofen
10	PU/PD 3 mo	None	Proteinuria	None	140	IMT	None	None
11	PU/PD, polyphagia 4 mo	↑SAP, ↑ALT, ↑BUN	Proteinuria	Lymphopenia	N/A	N/A	None	None
12	PU/PD (for 1 y)	↑BUN	N/A	None	180	Atopy	Mitral valve regurgitation	None
13	PU/PD	↑SAP, ↑ALT	None	None	105	Food atopy	Lameness	Cyclosporine
14	PU/PD for 3 y, weight gain	↑SAP, ↑ALT, ↑cholesterol	Microalbuminuria	Neutrophilia	145	Atopy	Hepatomegaly	Denamarine
15	Polyphagia 2 mo	None	None	None	N/A	None	Head and neck pain	Carprofen as needed
16	None	None	Proteinuria	None	N/A	None	Cardiomegaly	None
17	Polyphagia, weight gain 2 mo	↑SAP, ↑ALT^a^	N/A	Anemia	150	None	None	Prednisone+doxycycline
18	None	None	None	None	N/A	Food atopy IBD	None	Famotidine, fluoxetine hydrochloride
19	None	↑SAP, ↑ALT^a^	None	None	135	Atopy	Hepatomegaly	Prednisone
20	None	↑SAP, ↑cholesterol	Proteinuria	None	N/A	None	Cushing's disease	Trilostane
21	None	None	Proteinuria	None	135	Atopy KCS	None	None
22	None	None	N/A	None	N/A	None	None	None
23	None	↑SAP	None	None	N/A	Food atopy IBD	None	Carprofen as needed
24	Weight gain 2 mo	None	N/A	None	140	N/A	Hepatomegaly	None
25	PU/PD 2 mo	None	None	None	N/A	N/A	None	None
26	None	None	None	Leukopenia	115	N/A	Pancreatitis, seizures	None
27	PU/PD, polyphagia 2 mo	None	None	None	150	N/A	Collapsed trachea	None
28	None	↑SAP	None	None	180	Food atopy	None	None
29	Polyphagia 2 mo	↑SAP, ↑ALT	None	Neutrophilia	N/A	Food atopy	Hepatomegaly	None

ALT, alanine aminotransferase; IBD, inflammatory bowel disease; IMT, immune mediated thrombocytopenia; KCS, keratoconjunctivitis sicca (dry eye); N/A, data not available; PD, polydipsia; PU/PD, poldypsia/polyuria; PP, polyphagia; PU, polyuria; SAP, serum alkaline phosphatase; WG, weight gain; WNL, within normal limits.

a Treated with systemic steroids prior to laboratory analysis.

Serum analysis of SARDS patients revealed abnormalities in 55% of patients (16/29), with increase in SAP/ALT being the most frequent finding (Table 4). Urine analysis revealed proteinuria/microalbuminuria in 45% of patients (10/22; urine analysis data were missing for 7 patients; Table 4). Blood pressure evaluation revealed systemic hypertension in 21% of patients (4/19; in 10 this was not evaluated; Table 4). Systemic hypertension was classified as SBP equal to or higher than 160 mmHg. Historical presence of allergic and autoimmune diseases was documented for 59% of patients (13/22; Table 4). In 7 patients, owners were unsure about incidence of allergic and autoimmune diseases, or gave conflicting responses compared to information from medical records provided by referring veterinarians; these patients were not included in analysis of allergic and autoimmune disease incidence. Atopy (50%; 11/22) and food allergies (27%; 6/22) were the most frequently described allergic diseases (Table 4). Radiological abnormalities were observed in 38% of patients (11/29), with hepatomegaly (20.7%; 6/29) being the most frequent finding (Table 4).

None of the evaluated dogs had historical evidence of neoplastic disease, or evidence of neoplastic disease observed by thoracic and abdominal radiography and CT imaging.

### 3.9 The relation of systemic abnormalities to retinal detachment

Systemic abnormalities were more common in the RD group compared to the ND group; however, the difference between groups did not reach statistical significance by the Fischer's exact test, most likely due to the small sample size: elevated liver enzymes — SAP/ALT (RD = 64% (7/11); ND = 44% (8/18), *p* = 0.7; OR = 1.7); and proteinuria (RD = 75% (6/8); ND = 28.5% (4/14), *p* = 0.07; OR = 7.5). Systemic hypertension did not seem to be a contributing factor to retinal detachment (RD = 22.2% (2/9); ND = 20% (2/10), *p* = 1; OR = 1.1). There was no statistically significant difference in age (RD = 7.9 ± 1.9 years (mean±SD); ND = 7.6 ± 1.7 years, *p* = 0.69, Student's *t* test) or duration of blindness prior to presentation to an ophthalmologist (RD = 18 ± 7 days; ND = 21 ± 12 days, *p* = 0.28) between the RD and ND group.

### 3.10 Immunohistochemistry characterization of SARDS retinas

**Figure 10:**
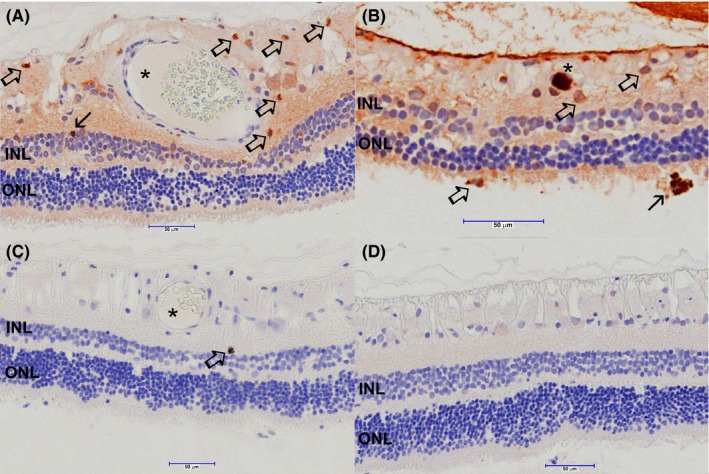
Immunohistochemistry (IHC) analysis of SARDS retina collected within 1 month after onset of blindness (7‐y‐old CM poodle). A, Anti‐CD3 antibody (T‐cell marker) staining shows presence of numerous T‐lymphocytes in the perivascular space (open arrows point to intensively labeled cells with typical lymphocyte morphology‐smaller cells with large nuclei almost completely filling the cellular space; star marks blood vessel lumen). Sporadically present strongly positive anti‐CD3 stained cells are present in the inner nuclear layer (closed arrow). B, Anti‐Ig cocktail antibodies were used for identification of plasma cells. Immunohistochemistry staining shows positive cells with typical plasma cell morphology (large cells with smaller nucleus and rather extensive cytoplasm staining shows presence of cells in the inner retina and subretinal space [open arrows]). In this particular retinal section, cells were in a few isolated regions of the peripheral retina. Extensive staining is present in the blood vessel lumen (star). Closed arrow points to the large macrophage (or transformed RPE cell) in the subretinal space (closed arrow). C, Anti‐CD79 antibody (B‐cell marker) staining shows presence of a single isolated positive cell in the perivascular space in this retinal section (open arrow). Blood vessel lumen is marked with a star. D, SARDS retina stained with omission of the primary antibody (negative control)

**Figure 11:**
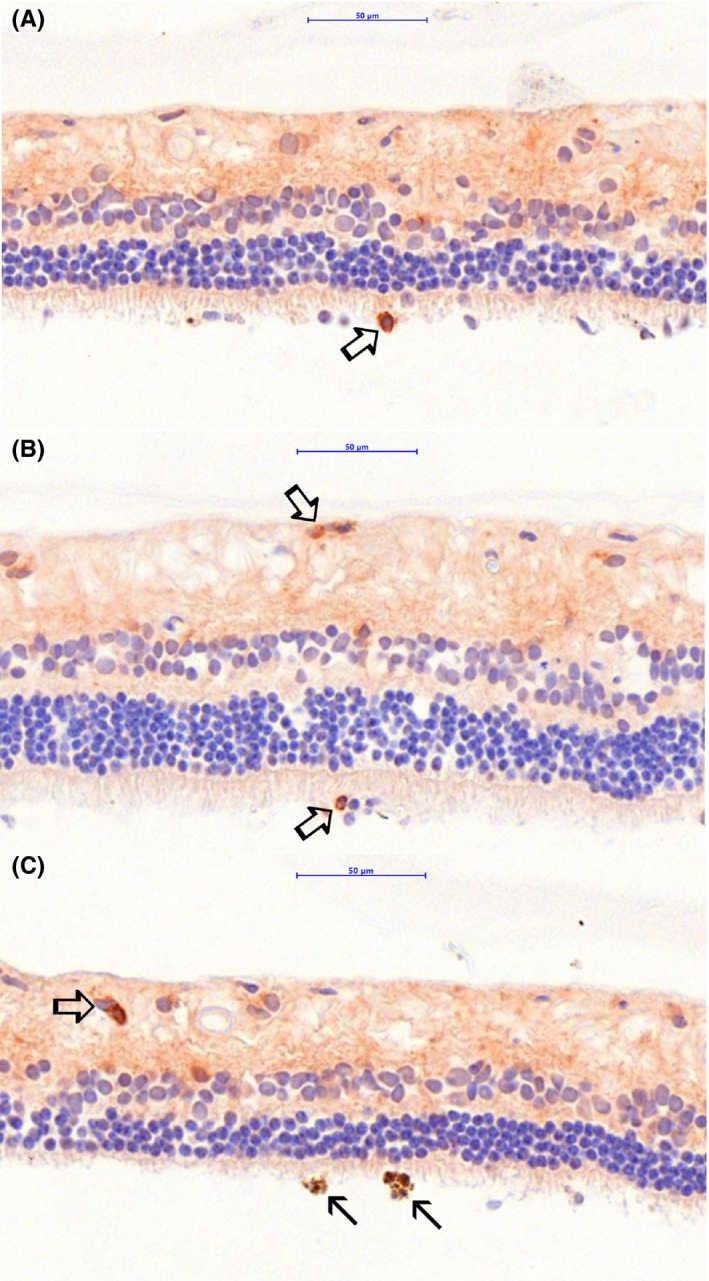
Immunohistochemistry (IHC) analysis of SARDS retina collected within 1 month after onset of blindness (7‐y‐old CM poodle). A, Anti‐CD3 antibody (T‐cell marker) staining shows presence of T‐lymphocytes infiltrating outer segments of photoreceptors (open arrow). B, Anti‐CD3 antibody (T‐cell marker) staining shows presence of T‐lymphocytes infiltrating outer segments of photoreceptors and nerve fiber layer (open arrows). C, Anti‐CD3 antibody (T‐cell marker) staining shows presence of T‐lymphocytes infiltrating inner retinal layers in the perivascular spaces. Closed arrows show infiltration of macrophage/RPE activated cells in the photoreceptor outer segment. Anti‐CD18 IHC was performed, however, no evaluated sections contained macrophage‐like cells infiltrating photoreceptor layer. Anti‐CD18 IHC did not label RPE cells

**Figure 12:**
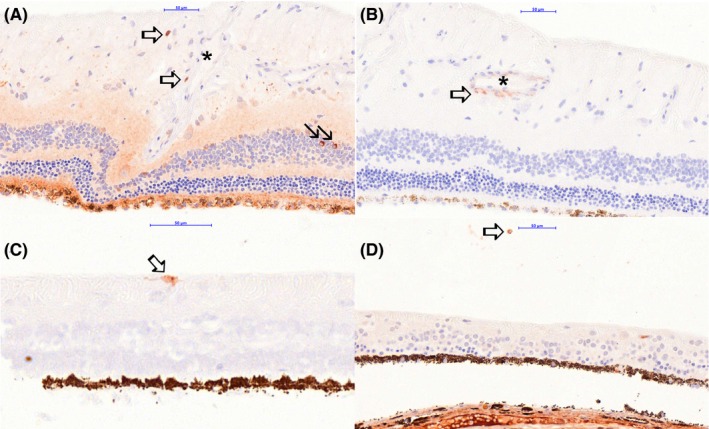
Immunohistochemistry (IHC) analysis of SARDS retina collected 19 month after onset of blindness (8‐y‐old CM maltese). A, Anti‐CD3 antibody (T‐cell marker) staining shows presence of sporadic T‐lymphocytes in the perivascular space (open arrows; star marks blood vessel lumen). Sporadically present strongly positive anti‐CD3 stained cells are present in the inner nuclear layer (closed arrow). B, Anti‐CD79 antibody (B‐cell marker) staining shows presence of IHC positive cells in the perivascular space in this retinal section (open arrow). Blood vessel lumen is marked with a star. C, Anti‐CD18 antibody (macrophage marker) staining shows presence of a single IHC positive cell in with characteristic macrophage morphology (open arrow). D, Anti‐Ig cocktail antibodies were used for identification of plasma cells. Immunohistochemistry staining shows positive cell with typical plasma cell morphology in the vitreal space (open arrow)

**Figure 13:**
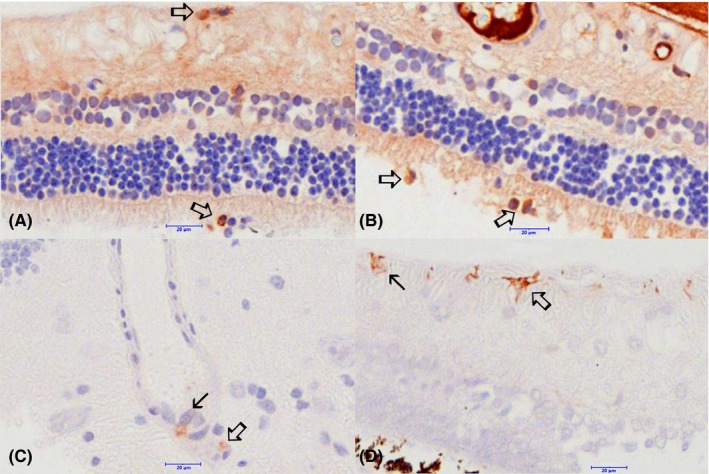
Immunohistochemistry (IHC) analysis of SARDS retinas with higher magnification. A, Anti‐CD3 antibody (T‐cell marker, 7‐y‐old CM poodle) staining shows presence of sporadic T‐lymphocytes in the sub retinal and pericapillary space (open arrows). B, Anti‐Ig cocktail antibodies were used for identification of plasma cells (7‐y‐old CM poodle). Immunohistochemistry staining shows positive cell with typical plasma cell morphology in the subretinal space (open arrow). C, Anti‐CD79 antibody (B‐cell marker, 8‐y‐old CM Maltese) staining shows presence of IHC positive cells in the perivascular space in this retinal section (open arrow), and presence of cells in the blood vessel lumen (closed arrow). D, Anti‐CD18 antibody (macrophage marker) staining shows presence of IHC positive cells with characteristic activated macrophage/microglia morphology (open arrow), and the less prominent branching of cellular processes in likely inactive macrophage/microglial cells (closed arrow)

**Figure 14:**
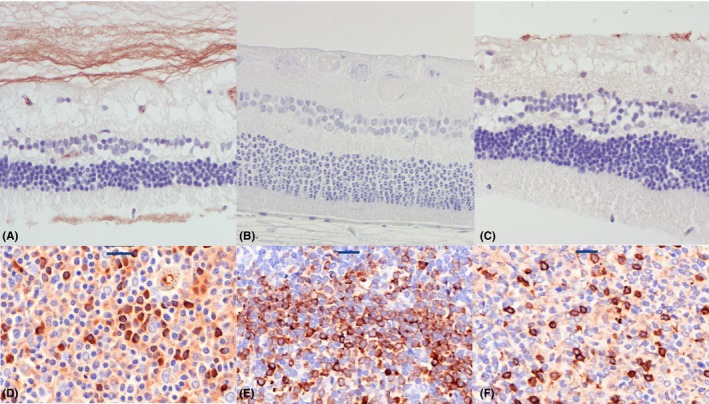
Control immunohistochemistry (IHC) analysis of healthy canine retinas and lymph nodes. (A and D) Anti‐Ig cocktail antibody (plasma cell marker) staining; (B and E) anti‐CD79 (B‐cell marker) staining; (C and F) anti‐CD3 (T‐cell marker) staining. Scale bar = 20 μm

Immunostaining analysis showed focal presence of immune cells in canine SARDS retinas (Figures 10, 11, 12, 13), which were usually located in perivascular (Figures 10, 12 and 13) and subretinal spaces (Figures 10, 11, 12, 13); there were no such changes in control healthy canine retinas (Figure 14). T‐cells were the predominant cell population identified in SARDS retinas. Evaluation of SARDS retina with relatively recent onset of blindness (Figures 10, 11 and 13), and SARDS retina 19 months after onset of blindness (Figures 12 and 13), revealed similar localization and frequency of immune cell populations, despite prolonged course of disease. We did not perform IHC grading of evaluated retinas, since retinal sections frequently had just a few focal regions of cellular infiltrates per evaluated slide.

### 3.11 Microarray analysis of canine SARDS retina

**Figure 15:**
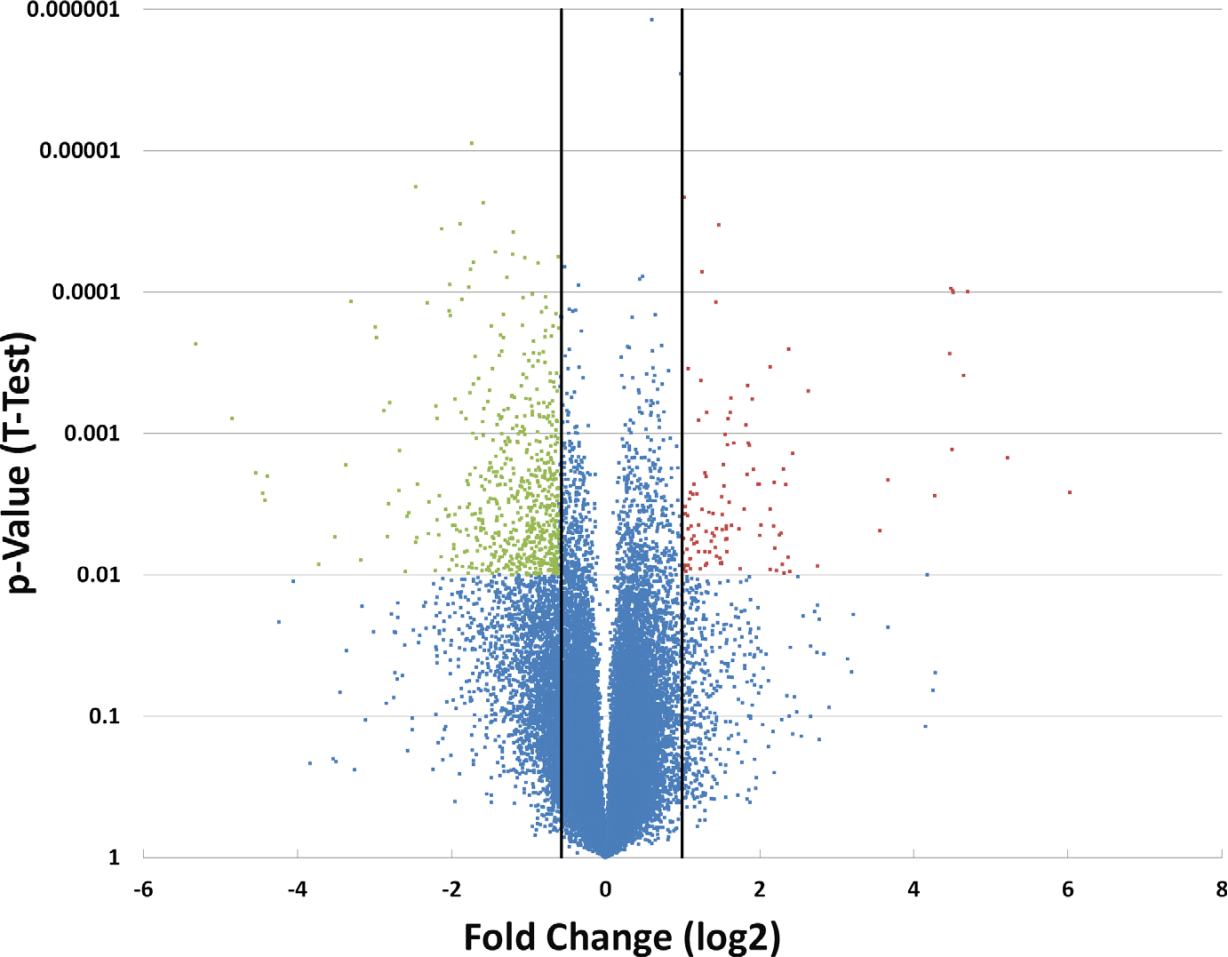
Volcano plot of gene expression data. Probe sets corresponding to expression at least two‐fold higher in SARDS retinas and with a *P*‐value below 0.01 are highlighted in red. Those with an at least two‐fold reduced expression in SARDS and a *P*‐value below 0.01 are marked green

Global gene expression analysis resulted in the identification of 252 genes represented by 348 probe sets that were more prevalent in the SARDS samples compared to control retinas. Conversely, 483 genes represented by 656 probe sets appeared to have reduced expression in SARDS retinas (Figure 15). Reduced expression was observed for a large number of functionally diverse genes (Table 5). However, many of these were related to visual transduction and structural photoreceptor components. Additional functional groups with lower expression in SARDS retinas included voltage gated channels and other molecules involved in ion transport. These changes were likely indicative of photoreceptor and neuronal loss, rather than regulation of gene expression.

**Table 5:** Functional clustering of genes with significantly decreased expression in SARDS retinas

Probe_ID	Gene	Controls	SARDS	Fold_Change	*q*‐value (%)
Phototransduction					
Cfa.1197.1.S1_s_at	Rhodopsin(RHO)	13.62	9.78	−14.26	0
CfaAffx.27956.1.S1_s_at	Phosphodiesterase 6A (PDE6A)	13.47	9.97	−11.32	0
CfaAffx.3368.1.S1_at	Guanylate cyclase activator 1B (GUCA1B)	12.84	9.72	−8.68	0
CfaAffx.25662.1.S1_s_at	Phosphodiesterase 6B(PDE6B)	13.46	10.62	−7.18	0
Cfa.3474.1.S1_s_at	G protein subunit alpha transducin 1(GNAT1)	10.52	8.01	−5.70	0
CfaAffx.3796.1.S1_s_at	Cyclic nucleotide gated channel alpha 1(CNGA1)	13.05	10.54	−5.69	0
CfaAffx.10557.1.S1_s_at	G protein‐coupled receptor kinase 1(GRK1)	9.53	7.10	−5.39	0
CfaAffx.26380.1.S1_at	Solute carrier family 24 member 1(SLC24A1)	9.61	7.32	−4.90	0
Cfa.3764.1.S1_at	S‐antigen visual arrestin(SAG)	14.10	11.86	−4.73	0
CfaAffx.13604.1.S1_s_at	Cyclic nucleotide gated channel beta 1(CNGB1)	8.30	6.11	−4.59	0
CfaAffx.27636.1.S1_s_at	Guanylate cyclase 2F, retinal(GUCY2F)	6.96	4.76	−4.58	0
Cfa.17905.1.S1_s_at	G protein subunit beta 1(GNB1)	12.17	10.10	−4.20	0
CfaAffx.20176.1.S1_s_at	Phosphodiesterase 8A(PDE8A)	9.81	7.80	−4.03	0
Cfa.3772.1.S1_s_at	Phosphodiesterase 6G(PDE6G)	13.88	11.88	−4.01	0
Cfa.3520.1.S1_s_at	Peripherin 2(PRPH2)	12.91	10.93	−3.94	0
Ion transport/gated channels					
CfaAffx.17709.1.S1_at	Potassium voltage‐gated channel subfamily B member 1(KCNB1)	8.94	6.36	−5.95	0
CfaAffx.3964.1.S1_at	Potassium voltage‐gated channel modifier subfamily V member 2(KCNV2)	11.38	9.37	−4.02	0
CfaAffx.9377.1.S1_s_at	Solute carrier family 4 member 7(SLC4A7)	8.46	6.61	−3.59	0
CfaAffx.19669.1.S1_s_at	Solute carrier organic anion transporter family member 4A1(SLCO4A1)	7.06	5.07	−3.97	0
CfaAffx.24373.1.S1_at	Calcium voltage‐gated channel subunit alpha1 F(CACNA1F)	6.70	5.15	−2.92	0
CfaAffx.12747.1.S1_s_at	Solute carrier family 12 member 6(SLC12A6)	9.71	8.71	−2.00	0

Values represent normalized log2 transformed signal intensities.

The *q*‐value predicts the fraction of false positive findings given the statistical parameters applied during the analysis.

In contrast, transcripts more abundant in the SARDS retina were overwhelmingly related to immune phenomena (Table 6). Our data revealed a very pronounced increase in transcripts encoding T, B, and natural killer (NK) cell markers. We also detected elevated transcript levels of immunoglobulin components, chemokines and their receptors, components of the complement cascade, and a host of other molecules. Taken together, our data suggest that SARDS is accompanied by immune‐cell infiltration into the retina in concert with active inflammation.

**Table 6:** Functional clustering of genes with significantly elevated expression in SARDS retinas

Probe_ID	Gene	Controls	SARDS	Fold_Change	*q*‐value(%)
Immunoglobulins					
Cfa.4556.3.A1_a_at	IgA heavy chain constant region (IGHAC)	5.39	11.42	65.03	0
Cfa.4465.2.S1_at	Ig lambda‐7 chain C region‐like (LOC608238)	7.80	12.50	26.01	0
CfaAffx.21065.1.S1_s_at	Immunoglobulin lambda‐like polypeptide 5 (LOC100687054)	7.98	12.50	22.85	0
CfaAffx.28248.1.S1_at	Ig heavy chain V region 3‐6‐like (LOC102153768)	5.61	8.83	9.29	0
CfaAffx.20171.1.S1_s_at	Low affinity immunoglobulin gamma Fc region receptor II (LOC100856270)	6.35	8.44	4.25	0
CfaAffx.15259.1.S1_at	Activated leukocyte cell adhesion molecule (ALCAM)	7.22	8.79	2.96	0
Antigen processing/presentation					
Cfa.182.1.S2_at	Major histocompatibility complex, class II, DQ alpha 1 (DLA‐DQA1)	7.97	10.25	4.86	0
CfaAffx.2152.1.S1_s_at	HLA class II histocompatibility antigen, DQ beta 2 chain‐like (LOC100856137)	8.02	10.22	4.59	0
CfaAffx.2126.1.S1_s_at	MHC class II DR alpha chain (DLA‐DRA)	9.80	11.93	4.39	0
CfaAffx.18761.1.S1_s_at	Cathepsin S (CTSS)	9.01	11.03	4.06	0
Cfa.18297.1.S1_at	Major histocompatibility complex, class II, DM alpha (DLA‐DMA)	9.16	10.74	2.98	0
CfaAffx.27739.1.S1_at	CD74 molecule (CD74)	8.86	10.38	2.87	0
Cfa.181.1.S1_at	MHC class II DLA DRB1 beta chain (HLA‐DRB1)	9.93	11.44	2.85	0
T/B/NK cell markers					
Cfa.3794.1.A1_s_at	T‐cell differentiation protein (MAL)	7.54	11.72	18.13	0
Cfa.15473.1.A1_at	Lymphocyte antigen 86 (LY86)	6.81	9.19	5.18	0
Cfa.14560.1.S1_at	CD48 molecule (B‐lymphocyte activation marker)	6.81	9.12	4.99	0
Cfa.14436.1.A1_at	Leukocyte‐associated immunoglobulin‐like receptor 1 (LAIR1)	7.15	9.29	4.40	0
Cfa.18829.1.S1_s_at	CD2 molecule (T‐cell surface antigen)	6.06	8.04	3.96	0
CfaAffx.30242.1.S1_at	CD53 molecule (CD53)	6.22	8.09	3.66	0
CfaAffx.6644.1.S1_s_at	T‐cell receptor beta‐1 chain C	6.83	8.52	3.23	0
Cfa.3629.2.S1_s_at	CD86 molecule (CD86)	8.38	9.82	2.72	0
Chemokine signaling					
Cfa.16327.1.S1_at	C‐C motif chemokine receptor 5 (CCR5)	4.86	7.30	5.40	0
Cfa.12237.1.A1_at	Chemokine (C‐C motif) ligand 23 (CCL23)	9.25	11.64	5.26	0
Cfa.16590.1.S1_s_at	Chemokine (C‐X‐C motif) ligand 10 (CXCL10)	6.41	8.43	4.06	0
Cfa.11063.1.A1_at	Adenylate cyclase 7 (ADCY7)	7.53	9.37	3.59	0
Complement Cascade					
CfaAffx.28463.1.S1_at	Complement C3 (C3)	9.16	11.93	6.85	0
Cfa.10921.1.S1_s_at	Complement C1q C chain (C1QC)	9.92	11.88	3.88	0
Cfa.16857.1.S1_at	Complement C1q B chain (C1QB)	9.40	11.07	3.18	0
Cfa.16469.1.S1_x_at	Serpin family A member 1 (SERPINA1)	5.81	7.38	2.98	0
CfaAffx.22561.1.S1_s_at	Complement C1q A chain (C1QA)	9.24	10.77	2.90	0
Cfa.16472.2.S1_a_at	Complement C1s (C1S)	7.06	8.31	2.38	0

Values represent normalized log2 transformed signal intensities.

The *q*‐value predicts the fraction of false positive findings given the statistical parameters applied during the analysis.

## 4 DISCUSSION

Sudden Acquired Retinal Degeneration Syndrome was first reported almost four decades ago; however, the precise etiology of the disease is still being extensively debated without a clear consensus on its exact nature.^1^ SARDS is most frequently seen in middle aged/older females of small breeds, with mixed‐breed dogs, Dachshunds, Pugs, and Miniature Schnauzers being the most frequently affected, which corresponds to our findings in this study.^2^, ^3^, ^24^, ^25^ Previous studies found a relatively high incidence of metabolic abnormalities, such as polyuria and polydipsia (38%,^2^ 30%^3^), polyphagia (19%,^2^ 20%^3^), and alkaline phosphatase elevation (37%,^2^ 28%^3^), which was also in good accord with our findings in similar percentages. The most recent study of a Canadian patient population, however, revealed much higher incidence of metabolic abnormalities in SARDS patients.^26^ This study confirmed relatively high incidence of proteinuria (45%) and systemic hypertension (21%) in SARDS patients, consistent with data from a previous study by Carter et al, and the most recent study by Leis et al,^26^, ^27^ Radiography evaluation revealed presence of hepatomegaly in 21% of patients, which was somewhat higher than previously reported (12%).^3^ Brain imaging did not find evidence of intracranial neoplasia, consistent with a previously published report.^28^

Detailed fundus evaluation in this study revealed numerous funduscopic abnormalities, with retinal vascular attenuation and pale optic nerve head appearance being the most frequently observed, followed by change in tapetal reflectivity. Similar findings have been reported, however, with relatively minor incidence of pale optic nerve head appearance.^2^ This is the first study to describe presence of frequently observed retinal inflammatory lesions, such as perivascular hyper‐reflective lesions, perivascular retinal edema, and potentially perivascular exudative lesions in SARDS patients.

This study effectively utilized SD‐OCT technology to demonstrate the relatively high incidence of retinal lesions (RD, focal retinal thinning, and chorioretinal scars) in SARDS eyes. These lesions were not easily detected by indirect ophthalmoscopy or review of images captured by fundus photography. Considering that almost all patients were evaluated within the first 4 weeks after onset of blindness, data from this study can serve as possible evidence of early retinal changes in SARDS eyes, before more advanced retinal degenerative changes begin to develop. Careful analysis of retinal OCT scans revealed predominantly perivascular localization of lesions with inflammatory appearance, which was also confirmed by histopathological analysis of limited retinal tissue specimens. An equally important finding of this study was existence of photoreceptor loss, outer segment disorganization, and loss of IS‐OS junction in all evaluated SARDS patients. These have been previously described as the most frequently observed features in the human npAIR patient population.^29^, ^30^, ^31^, ^32^

This study demonstrated relatively high incidence of submillimeter RD in SARDS eyes, which did not seem to be a result of systemic hypertension. Analysis of systemic parameters revealed higher incidence of systemic abnormalities in the RD group compared to the ND group, with proteinuria being the most frequently observed anomaly. Additional studies will be needed to further elucidate the significance of these findings. Considering that proteinuria may result from intermittent systemic hypertension,^33^, ^34^ it cannot be absolutely ruled out that observed perivascular RDs are not the result of intermittent episodes of high blood pressure that may damage the capillary bed, with consequent exudation and retinal detachment, as frequently seen in dogs with systemic hypertension.^35^, ^36^ Retinal funduscopic changes in this study had many features similar to those previously described in monkeys and humans with chronic systemic hypertension.^37^ These changes include narrowing and pseudo‐narrowing of retinal arterioles, increased tortuosity of arterioles, possible zones of focal transudates, possible inner retinal ischemic spots/cotton‐wool spots, retinal edema and cystoid retinal degeneration with possible subretinal transudate, and fibrosis.^37^

As a result of hypertensive choroidopathy, a serous RD may develop,^37^ which was the most frequently observed OCT feature in this study. There is a possibility that more subtle intensity of systemic hypertension with transient periods of higher blood pressure may result in the milder profile of retinal changes (as observed in this study), which would explain the presence of retinal changes in patients with normal blood pressure values. While systemic hypertension hypothesis is plausible, and is supported by many of the funduscopic features in SARDS retinas, there is a strong line of contradictory evidence from this study. Herring et al showed evidence of retinal hemorrhages in 20% of systemically hypertensive diabetic dogs, where systemic hypertension was defined as an SBP value above 150 mm Hg, and study by Leblanc et al reported a 40% incidence of retinal hemorrhages in systemically hypertensive dogs, where systemic hypertension has been defined as an SBP value of 160 mm Hg (or higher).^35^, ^36^ In this study, incidence of retinal hemorrhages was extremely low (1/29; 3.4%).

Limited histological data and some OCT lesions from this study support the notion of the inflammatory nature of lesions with cellular exudates in subretinal spaces, indirectly ruling out the possibility of a transudative process, as observed in hypertensive choroidopathy.^37^ In a 2008 study, we presented histological evidence of perivascular T and B‐cell inflammatory exudates, which could potentially explain the relatively frequent presence of RDs and primary photoreceptor lesions in perivascular regions observed by OCT imaging.^5^ These findings were confirmed in this study as well, further reinforcing the theory of the immune‐mediated nature of observed retinal changes. The retinal nerve fiber layer has increased sensitivity to damage associated with systemic hypertension, due to the development of RNFL, and resulting optic nerve capillary damage from regional RNFL loss or severe optic nerve damage.^37^ In our previous study, we showed that retinal thinning is a feature observed in SARDS patients.^5^

In this study, we did not find evidence of RNFL loss in the superior or inferior retina, or in the peripapillary region. Furthermore, all evaluated patients had complete pupillary light reflexes with blue light illumination, further providing evidence of relatively preserved functional optic nerve properties. A possible reason for the observed discrepancy between the data from our previous study and this one could be that many imaged SARDS dogs had blindness for a longer period of time in the 2008 study, which could have resulted in more advanced retinal damage and development of RNFL thickness deficits. However, the more likely explanation for the discrepancy in RNFL thickness results is the fact that the technology differed between studies. That used in our 2008 study likely included the inner plexiform and inner nuclear layer in the RNFL thickness calculation, compared to much more sophisticated imaging routines used in this study. In our previous study, documented values for RNFL thickness in the *area centralis* of control healthy dogs were close 96 μm, while in this study, observed values were 26 μm, clearly suggesting that previous delineation routines did not calculate only RNFL thickness. Considering that data in this study showed localized regions of severe retinal structural loss, we cannot exclude the possibility that previously observed RNFL thinning was actually a result of the erroneous incorporation of different retinal layers in calculated values. Since OCT technology used in this study has dramatically better resolution, we now consider that RNFL does not seem to be damaged in SARDS retinas.

In this study, we attempted to characterize molecular events associated with SARDS retinal damage in dogs, with the goal of better understanding the pattern of morphological changes observed with OCT imaging. These data are the first to describe detailed changes in the gene expression pattern, and potential immunological consequences in a species with large eyes and spontaneously occurring disease similar to npAIR in humans. The major limitation of this study is that number of analyzed SARDS samples was quite small, and tissue samples for used microarray analysis were collected from deceased dogs after prolonged duration of blindness. While these factors likely influenced the expression levels of some genes, the overall similarity between immunoglobulin and complement gene expression between this study and our previously reported data from a single SARDS retina sample collected in early days after blindness development,^5^ suggests that observed gene expression changes may indeed adequately reflect the immune‐mediated processes present in SARDS retina at different stages of disease. Furthermore, IHC data are indicative of the unabated immune response in SARDS eyes regardless of disease duration (Figures 10 and 12), providing more supporting evidence of the relevance of presented microarray data in this study.

Among genes with elevated expression levels in SARDS retinas, the preponderance of genes mediating various aspects of immune‐mediated response was striking. Prominent functional categories of genes with elevated expression in SARDS retinas include antigen presentation, complement activation, leukocyte activation and adhesion, lysosomal and proteasome activity, and immunoglobulin production. In addition, numerous genes with a function in apoptosis and inflammation signaling are more abundant in SARDS retinas. It must be noted that many of the identified genes with altered expression levels are associated with several biologic functions; thus inclusion in one functional category does not exclude a gene's involvement in additional molecular pathways.

Our analyses also indicate that SARDS leads to lower expression levels for a large number of genes, primarily those associated with photoreceptor function. Reduced mRNA levels of individual genes could partially result from transcriptional control mechanisms; however, the more likely explanation is that observed changes are a result of primary photoreceptor damage and apoptotic loss in SARDS eyes, further supporting the OCT and histology data presented in this study. These findings are consistent with the previous report by Miller et al, who showed massive apoptosis of photoreceptors in SARDS retinas.^8^

The systematic comparison of gene expression findings in this study to those presented previously by other investigators is not straightforward, due to the different formats of gene arrays used, selection of genes represented on each array, incomplete identification of orthologs between animal species, types of disease investigated, and methodology used for evaluation of RNA expression.^38^ Previous studies had observed a significant decrease in expression levels for several photoreceptor genes in canine models of retinal degeneration.^39^, ^40^ Our own studies showed significant upregulation of genes associated with antigen presentation, complement activation, lysosomal and proteasome activity, and apoptosis in canine glaucomatous retinas, raising the possibility that many of observed changes in SARDS retinal gene expression may be a result of the reactive immune system cell response to neuronal death in the retina.^23^ Similar findings have also been reported by evaluating gene expression of inflammatory genes in different canine models of hereditary retinal degeneration.^41^ However, findings of increased immunoglobulin gene expression, increased expression of leukocyte adhesion molecules and T‐cell differentiation gene (MAL), coupled with immunohistochemistry evidence of T‐cell, plasma cell/B‐cell, and macrophage presence in canine SARDS retina may be indicative of the primary immune system mediated nature of retinal insult. Interestingly, observed gene expression changes and presence of different immune cell populations in SARDS retinas collected within 1 month^5^ and 8‐30 months after onset of blindness showed striking similarities, potentially supporting the notion of unabated and continuous immune‐mediated insult on SARDS retina independent of blindness duration. An obvious limitation of this study remains the lack of RT‐PCR and western blot experiments to confirm observed gene expression changes.

While different hypotheses have been proposed on the etiology of SARDS, there is still no consensus on the exact nature of this syndrome, despite many clinical findings suggesting it is extremely similar to non‐paraneoplastic autoimmune retinopathy in humans.^1^ The biggest obstacle to a more precise determination of SARDS etiology has been the lack of studies evaluating detailed histological, molecular, and morphological features of early retinal changes in SARDS patients.^5^, ^8^ Considering that SARDS does not result in painful eyes, access to retinal tissue has traditionally been very limited, seriously impeding the possibility of performing studies on tissues from patients in the early stage of disease.

The frequent presence of endocrine and metabolic abnormalities in this patient population was used to raise speculations toward a possible toxic, endocrine, or neuroendocrine etiology, while limited histological and molecular evaluation has revealed the presence of photoreceptor apoptosis with a possible suggestion of a steroid‐induced toxic form of retinal damage.^8^ However, the limited histological and molecular data available from several studies revealed presence of serum retinal autoantibodies, increased complement activation, presence of immunoglobulin producing B‐cells, presence of T‐cells, and upregulation of genes mediating increased immune and inflammatory response in SARDS eyes, potentially suggestive of the autoimmune nature of the disease.^5^, ^6^, ^7^ Considering the widespread depression of retinal electrical activity, which does not correspond to funduscopic retinal changes or OCT structural deficits, we speculate that the potential target of the primary immune insult in SARDS eyes is the retinal pigment epithelium (RPE). It has recently been demonstrated that autoantibodies in melanoma‐associated retinopathy recognize TRPM1 and TRPM3 cation channel proteins expressed on melanocytes, bipolar retinal neurons, and RPE cells.^42^

The autoimmune hypothesis of SARDS remains controversial in the veterinary ophthalmology community due to lack of traditional inflammatory changes during ocular examination, lack of serum retinal autoantibodies, and the well‐established fact that retinal autoantibodies can be detected in the serum of patients with many different neurodegenerative retinal diseases as a result of the reactive immune response to dying retinal cells.^8^, ^28^ Serum retinal autoantibodies are considered a hallmark of autoimmune retinopathies (AIR) in human patients, which are also characterized by sudden onset of blindness, severe decrease or absence of retinal electrical activity, and relatively normal fundus appearance.^43^, ^44^

This study found a relatively high incidence of potentially inflammatory lesions such as RDs, subretinal exudate, chorioretinal scars, focal perivascular retinal degenerative changes, and cystic retinal changes, which are almost classic OCT features of inflammatory retinal diseases. Detailed analysis of SD‐OCT images and limited histological data is highly suggestive of focal inflammatory insults resulting in exudative lesions, focal chorioretinal scars, and focal inflammatory lesions in different retinal regions. The result is primarily damage to photoreceptors; however, in some regions, outer plexiform and inner nuclear layers were also affected. Detection of immune cells or activated RPE cells in the region of insult can be a result of the primary inflammatory process, but could also derive from a reactive response to RD and subsequent focal retinal degeneration. Gene expression and immunohistochemistry data presented in this study are suggestive of the different inflammatory and immune‐mediated processes in SARDS retinas, further re‐enforcing potential immune‐mediated nature of retinal lesions observed.

In conclusion, observed morphological and molecular retinal changes in different stages of SARDS are highly suggestive of the immune‐mediated nature of retinal damage, with many OCT structural similarities previously observed and described in human npAIR patients. Our recent data described prevention of vision loss progression in SARDS dogs treated with aggressive immunosuppressive therapy, further enhancing the notion of the immune‐mediated nature of this disease, and the need for early therapeutic intervention before complete loss of vision develops (Grozdanic et al, Abstract no 82, American College of Veterinary Ophthalmology Annual Conference, Monterey, CA, October 2016). Proper classification of SARDS as an immune‐mediated disease may allow more aggressive and effective therapeutic approach for SARDS patients.
